# Processing Speed Mediates the Longitudinal Association between ADHD Symptoms and Preadolescent Peer Problems

**DOI:** 10.3389/fpsyg.2017.02154

**Published:** 2018-02-13

**Authors:** Anders L. Thorsen, Jocelyn Meza, Stephen Hinshaw, Astri J. Lundervold

**Affiliations:** ^1^Department of Biological and Medical Psychology, University of Bergen, Bergen, Norway; ^2^Department of Psychology, University of California, Berkeley, Berkeley, CA, United States; ^3^Department of Psychiatry, University of California, San Francisco, San Francisco, CA, United States; ^4^K.G. Jebsen Center for Research on Neuropsychiatric Disorders, University of Bergen, Bergen, Norway

**Keywords:** ADHD, inattention, processing speed, peer problems, mediation

## Abstract

We investigated the relation between dimensional aspects of inattention and hyperactivity-impulsivity in childhood and peer problems 4 years later, as well as the potential mediating effects of intellectual function. The sample included 127 children (32 with attention-deficit/hyperactivity disorder). Symptoms of inattention and hyperactivity-impulsivity were assessed via parent and teacher reports on Swanson Nolan and Pelham-IV questionnaire. Peer problems were assessed by parent reports on the Strengths and Difficulties Questionnaire, and children's intellectual functioning by the third edition of the Wechsler Intelligence Scale for Children. Linear regressions showed a significant effect of inattention on future peer problems, partially mediated by slow processing speed. These effects remained significant when ADHD status was covaried. Findings highlight the importance of processing speed in explaining the predictive relation between childhood inattention and later peer problems. Inattention and processing speed in early childhood are potentially malleable factors influencing adolescent social functioning.

## Introduction

Attention-deficit/hyperactivity disorder (ADHD) is a common childhood psychiatric disorder (American Psychiatric Association, 2013), with an estimated worldwide prevalence of 5–7% (Polanczyk et al., 2007). ADHD is characterized by developmentally extreme symptoms of inattention (IA) and/or hyperactivity-impulsivity (HI) that impair functioning in at least two settings (American Psychiatric Association, 2013). ADHD has been linked to a wide range of negative life outcomes, including problems related to family interactions (Peris and Hinshaw, 2003), social functioning with peers (Spira and Fischel, 2005; Hoza, 2007; Molina et al., 2009), and academic and vocational underperformance (Loe and Feldman, 2007; Kent et al., 2011; Hechtman et al., 2016). Several community-based studies have shown that even when below diagnostic thresholds, symptoms of IA and HI predict mental and physical health outcomes (e.g., Holmberg and Bölte, 2014). Moreover, intellectual functioning has been proposed to help explain how IA and HI relate to important life outcomes (McQuade and Hoza, 2008; Thaler et al., 2013). Still, there is a lack of longitudinal research including both symptom dimensions of IA and HI and intellectual function as predictors of future preadolescent peer problems.

### Peer problems and symptoms associated with ADHD

Peer problems have been extensively studied in children with ADHD (Hinshaw and Melnick, 1995; Hoza et al., 2005; Hoza, 2007; Lee et al., 2008; McQuade and Hoza, 2008; for a thorough review see Ros and Graziano, 2017). Difficulties with peers have been associated with impaired functioning across a wide range of domains (Wheeler and Carlson, 1994; Mrug et al., 2012). Research suggests that both IA and HI symptoms may influence peer relations, but in a different manner. Specifically, children with a combined presentation of ADHD (ADHD-C; i.e., those with a high number of inattentive and hyperactive-impulsive symptoms) are described as more aggressive with their peers than children in comparison groups, and therefore more likely to receive peer rejection, whereas children with primarily IA symptoms (ADHD-I) are often withdrawn during social interactions, probably related to a poor ability to recall the content of conversations with their peers along with other social skills deficits (Mikami et al., 2007). Furthermore, a cascading effect may exist (see Hinshaw, 2017), through which ADHD symptoms and social problems magnify each other over time (Tseng et al., 2014). Community studies show that these findings are not restricted to children with a formal ADHD diagnosis (e.g., Andrade and Tannock, 2014). However, questions remain regarding pathways between the IA and HI dimensions and future peer-related outcomes.

### Intellectual function and ADHD symptoms

Intellectual function is a multidimensional concept that has strong predictive power to important life outcomes in the general population (Deary, 2012). Tests of intellectual function, like the third edition of the Wechsler Intelligence Scale for Children (WISC-III; Wechsler, 1991), include subtests assessing partially independent indices of cognitive function (Kamphaus et al., 1994; Keith and Witta, 1997; Roid and Worrall, 1997). The WISC-III generates indices for Verbal Comprehension Index (VCI), Perceptual Organization Index (POI), Freedom from Distractibility Index (FFDI), and Processing Speed Index (PSI). Although a meta-analysis by Frazier et al. (2004) found a general pattern of impairment across these four indices, other studies have shown that IA may be particularly tied to impaired performance in specific aspects of intellectual function. For example, children with ADHD-I show slower processing speed compared to individuals with other presentations of ADHD (Chhabildas et al., 2001; Calhoun and Mayes, 2005; Riccio et al., 2006; Mayes et al., 2009; Thaler et al., 2013). Similarly, slow processing speed has been linked with teacher reports of hypoactivity, a behavior closely related to IA (Lundervold et al., 2011). This pattern suggests that there is a unique link between symptoms of IA and processing speed, which might aid the understanding of future problems with peers.

### Predictors of peer problems

Several factors may act as predictors of peer problems. In a community study, Bellanti and Bierman (2000) showed that both IA and intellectual function in kindergarten were independent predictors of social functioning in first grade. Interestingly, the association with poor social function was stronger for IA than for low intellectual ability. The importance of IA was also supported by Huang-Pollock et al. (2009), who showed that effects on social function (i.e., the ability to pick up subtle verbal cues and remember a conversation) were primarily driven by IA rather than HI symptoms. Others, like Bunford et al. (2015), have instead described IA as a mediator between specific aspects of intellectual/executive function (i.e., response inhibition) and social adjustment. Finally, as noted above, compelling findings show that children with ADHD-I show slow processing speed, and that this slowness can help explain why inattentive children struggle socially (Calhoun and Mayes, 2005; Thaler et al., 2013).

Taken together, previous research has documented a wide range of factors predicting problems with peers. Investigations including performance on a test of intellectual function point to the importance of processing speed, but there are still few population-based studies that investigate how different aspects of intellectual function might contribute to explain the link between IA and HI on future peer problems.

### The current study

In a clinically diverse sample of children participating in a population-based study, we first investigated the independent contributions of IA and HI in predicting future peer problems. IA and HI were reported by parents and teachers when the children were 7–9 years (baseline), and peer problems were reported by parents at baseline as well as when the children were in early adolescence (ages 11–13 years). Key demographic variables (age and sex), the presence of an ADHD diagnosis, and baseline peer problems reported by parents were adjusted for in the statistical analyses. Second, we investigated whether the association between IA/HI and peer problems was mediated by the child's performance on the key indices of the WISC-III at ages 8–10. Based on previous findings, we hypothesized that (i) IA and HI in primary school children would predict later peer problems over and above demographic variables and baseline peer problems; (ii) indices from WISC-III would partially mediate the direct effect of IA and/or HI on peer problems, with the strongest mediating effect from processing speed, given its prior linkages with IA; and (iii) that these findings would be retained with the presence of an ADHD diagnosis included as a covariate.

## Methods

### The bergen child study

The Bergen Child Study (BCS) is a longitudinal, multi-wave, population-based study on childhood mental health and development. The present study includes data from three sequential time points of data collection. The first study wave was launched in 2002, organized into three phases. In the first phase, a questionnaire including the Strength and Difficulties Questionnaire (SDQ, Goodman, 1999) and the Swanson Nolan and Pelham-IV Questionnaire (SNAP-IV, Swanson, 1992) was sent to parents and teachers of all children attending 2nd−4th grade (7–9 years of age) in any school in the city of Bergen, Norway (9,439) (see Heiervang et al., 2007 for details). This constitutes the baseline measure of peer problems, as well as IA and HI symptoms, for the present study.

In a second phase, parents of children categorized as screen positives (for whom parents or teachers reported an SDQ total score that exceeded the 90th percentile and severe impairment on the impact section, or the score of one of the other included questionnaires exceeded the 98th percentile) and parents of a subset of screen negative children were interviewed according to the Development and Well-Being Assessment (DAWBA; Goodman et al., 2000). About 1 year later, a subgroup (*n* = 421) of the children and their parents were invited to a third clinical phase, including all children with any diagnosis according to DAWBA (*n* = 139). A total of 329 children and their parents participated in this phase, consisting of a short physical examination, a neuropsychological assessment including a test of intellectual function (WISC-III, Wechsler, 1991), and a clinical interview according to the Schedule for Affective Disorders and Schizophrenia for School Aged Children, Present and Lifetime Version (KSADS-PL; Kaufman et al., 1997) (see Lundervold et al., 2011 for details). We include this latter portion of the third phase as the intermediate time point, in between documentation of ADHD symptoms and later ascertainment of peer functioning, at which measures of intellectual functioning were ascertained.

Finally, when the children were 11–13 years old, parents and teachers completed a similar questionnaire as in the first wave, including the SDQ and SNAP-IV. This data collection represents the third time point.

The present study included the subsample of 127 children with complete data for the variables of interest (i.e., across all the three described time points). Approximately half of the mothers and fathers of the participating children had completed education at a college or university level (see Supplementary Table 1 for details). Information about education was not available for four fathers. Ethnicity was not assessed, given the homogeneity of the Norwegian population. That is, the vast majority of the population-level BCS sample spoke Norwegian at home, with only 5% reporting another primary language (Heiervang et al., 2007). The included subsample was not significantly different from those without complete data in terms of IA and HI scores, scores on the WISC-III, age, or sex (all *p*s > 0.05). Parents gave written consent for participation, and the study was approved by the Regional Committee for Medical and Health Research Ethics in Western Norway.

### Measures

*Peer problems* were assessed by the total sum score of parent reports on the peer problems subscale of the SDQ (Goodman, 1999), both at the first and third data points. Although cross-informant information from both parents and teachers is recommended (e.g., Renk and Phares, 2004), parent reports were selected as the outcome measure because teacher reports were available for only 66 participants (52%) at age 11–13. The subscale consists of five items rated on a Likert scale from 0 (“not true”) to 2 (“certainly true”), assessing whether the child is a victim of bullying, has at least one good friend, is solitary, is generally liked by other children and if he/she gets along better with adults than with other children. The items assessing the presence of at least one good friend and being liked by other children were reverse scored, so that a higher total score reflects more peer problems. The peer problems subscale has adequate psychometric properties and has been validated in several countries, including Nordic countries (Obel et al., 2004). The peer problems subscale of the SDQ had acceptable internal consistency at both data points (α = 0.72 at age 7–9; α = 0.79 at age 11–13).

*IA and HI* were assessed using the mean parent and teacher scores as reported on the SNAP-IV (Swanson, 1992) at the first data point (i.e., when the children were between 7 and 9 years old). The scale has been shown to have acceptable internal consistency (Bussing et al., 2008). It includes the 18 items used to define the IA and HI symptoms associated with an ADHD diagnosis. In the original SNAP-IV, each item is evaluated according to four levels. In the present study, parents and teachers evaluated the responses on a 3-level Likert-type scale (not true, somewhat true, or certainly true) to follow the response pattern of the remaining scales included in BCS questionnaires, with a higher score revealing higher symptom severity. Parent and teacher ratings were available for both included subscales. These different-source ratings were strongly correlated both for IA, *r*_(125)_ = 0.64, *p* < 0.01, and HI, *r*_(125)_ = 0.58, *p* < 0.01. Therefore, the mean scores reported by the parents and teachers were summed to construct cross-informant composite scores of IA and HI. To run an extra check for cross-informant discrepancy in predicting parent-rated peer problems, separate reports by source were included in supplementary analyses.

*Intellectual function* was assessed by the four indices generated from the Norwegian version (Eilertsen and Johnsen, 2003) of the WISC-III (Wechsler, 1991): the VCI, POI, FFDI, and PSI. The WISC-III was the most recent version available when the study was conducted, and the test has been validated in both typically developing and clinical samples (Burton et al., 2001). The difference between performance on similar indexes in WISC-III and WISC-IV is shown to be modest (Mayes and Calhoun, 2006). In the present study, the subtests of WISC-III were scored according to Swedish norms (Sonnander et al., 1998). The index scores were defined according to Kaufman (1994). VCI includes a set of verbal tasks assessing different aspects of the child's verbal reasoning abilities; POI includes timed tests of visual construction and analysis assessing the child's non-verbal reasoning abilities; FFDI comprises tasks assessing different aspects of working memory, and PSI includes tests assessing the speed with which the child is able to process information without errors.

*ADHD diagnosis*, along with fulfillment of criteria for any other DSM-IV disorder, was assessed using the K-SADS (Kaufman et al., 1997). The K-SADS is a structured clinical interview, validated for children between the ages of 6 and 18. Good agreement between K-SADS and other instruments used to identify ADHD was shown in a previous study from the BCS group (Posserud et al., 2014). Here, a group of psychologists and a medical doctor interviewed the parents and children to determine the presence or absence of any DSM-IV diagnoses. ADHD status was defined as positive if a child presently had a possible or definite diagnosis of ADHD. All other children were defined as ADHD negatives.

### Data analytic plan

Pearson correlations were calculated to assess covariation between all included variables. We then performed a series of *t*-tests to investigate group differences between children with positive and negative ADHD status and between boys and girls. Hierarchical linear regression analyses were then performed to investigate the contribution of each predictor variable, as well as each step's incremental explained variance. Age, gender, and ADHD status comprised in the first step, baseline peer problems the second step, and the parent/teacher composite scores of IA and HI symptoms the third step. The unique contribution of IA and HI was also tested by including one before the other in two different steps (see Pedhazur, 1982). Before investigating the indirect effect of the WISC indices on the direct effect between the two symptom dimensions (IA and HI) and later peer problems, we computed the least-angle regression procedure (LARS) in Stata 20 to reduce the number of variables (Efron et al., 2004). This restricted model selection approach introduces less noise and has greater power compared to a model including all variables. All variables from the previously described analyses were subjected to LARS, together with VCI, POI, FFDI, and PSI from the WISC-III. The selected variables were then included in a second set of regression analyses to investigate the significance of the WISC-III indices in this optimized model.

To confirm the effects revealed by these analyses, the variables from the restricted model were included in a parallel mediation analysis using the PROCESS macro (Hayes, 2013). This analysis evaluated the best-fitting WISC-indices (measured at age 8–12) as potential parallel mediators of the relation between childhood ADHD symptoms (at age 7–9) and preadolescent peer problems (at age 11–13), with baseline peer problems included as a covariate. The PROCESS analyses applied bias-corrected and accelerated (BcA) bootstrapping with 10,000 resamples, as this method has been shown to produce valid confidence intervals and reduce the influence of bias from non-normality and heterogeneity (Efron, 1987; Wilcox, 2012). All analyses except LARS were performed using IBM SPSS Statistics 23.

## Results

### Descriptive analyses and correlations

The total sample included 127 children, with 32 (25%) meeting criteria for an ADHD diagnosis (*n* = 5 females, 15%). Twenty-eight children with ADHD also met criteria for another diagnosis (*n* = 11 females, 39%). Ninety-five children did not meet criteria for ADHD (*n* = 40 females, 42%), but 29 of them met the criteria for one or more other mental disorders according to the KSADS-PL (Supplementary Figure 1).

The total sample included 82 boys (65%) and 45 girls, and boys were more likely to meet criteria for an ADHD diagnosis (χ = 7.34, *p* = 0.01). Boys showed higher scores on the IA and HI subscales, and scored lower on the FFDI and PSI indices, compared to girls (all *ps* < 0.05). Children with ADHD were older, obtained higher scores on the IA and HI scales, had higher peer problems scores, and obtained lower scores on VCI, POI, FFDI and PSI indices, compared to children without ADHD (all *ps* < 0.05; see Table 1).

**Table 1:** Age, SNAP subscales, and intellectual function.

	**Total sample (*N* = 127)**		**ADHD (*n* = 32)**		**Non-ADHD (*n* = 95)**		***p***	**Cohen's *d***
	**Mean**	**SD**	**Mean**	**SD**	**Mean**	**SD**		
Age	9.42	0.98	9.83	0.97	9.28	0.94	0.005	0.58
SNAP parent IA T1^a^	4.24	4.58	9.28	3.80	2.55	3.44	<0.001	1.86
SNAP parent HI T1^a^	3.29	4.34	7.09	5.34	2.01	3.03	<0.001	1.70
SNAP teacher IA T1^a^	3.93	4.91	9.63	4.58	2.01	3.25	<0.001	1.92
SNAP teacher HI T1^a^	3.20	4.82	7.13	5.74	1.88	3.64	<0.001	1.09
SDQ parent peer problems T1	1.69	2.01	2.69	2.15	1.36	1.86	0.001	0.66
SDQ parent peer problems T2	2.25	2.32	3.34	2.60	1.88	2.10	0.006	0.62
Full Scale IQ	89.16	17.86	80.72	20.78	94.09	16.17	0.002	0.72
VCI	89.44	15.92	82.78	16.08	91.68	15.31	0.006	0.57
POI	95.68	18.58	88.06	22.65	98.24	16.35	0.024	0.52
FFDI^a^	93.91	18.92	81.00	15.90	98.26	17.91	<0.001	1.01
PSI^a^	92.58	18.68	82.81	17.66	95.87	17.92	<0.001	0.73

a *Boys scored higher than girls, p < 0.05. ADHD, Attention-Deficit/hyperactivity Disorder; FFDI, Freedom from Distractibility; HI, Hyperactive-Impulsivity; IA, Inattention; POI, Perceptual Organization Index; Index; PSI, Processing Speed Index; SDQ, Strengths and Difficulties Questionnaire; SNAP, Swanson Nolan and Pelham-IV Questionnaire; VCI, Verbal Comprehension Index*.

The IA and HI subscales correlated moderately with peer problems and WISC-III indices, and the correlation between the peer relations scores in childhood and preadolescence was strong. Smaller, but statistically significant correlations were found for age, parent and teacher ratings of IA and FFDI (Table 2).

**Table 2:** Correlations between the included variables.

		**1**	**2**	**3**	**4**	**5**	**6**	**7**	**8**	**9**	**10**
1	Age	-									
2	Parent IA	0.21^*^	-								
3	Parent HI	0.09	0.73^**^	-							
4	Teacher IA	0.20^*^	0.64^**^	0.49^**^	-						
5	Teacher HI	0.07	0.48^**^	0.58^**^	0.72^**^	-					
6	Peer problems 7–9 year	0.12	0.56^**^	0.34^**^	0.29^**^	0.15	-				
7	Peer problems 11–13 year	0.06	0.45^**^	0.28^**^	0.38^**^	0.23^**^	0.49^**^	-			
8	VCI	−0.06	−0.32^**^	−0.20^*^	−0.40^**^	−0.25^**^	−0.20^*^	−0.12	-		
9	POI	0.02	−0.27^**^	−0.22^*^	−0.39^**^	−0.33^**^	−0.16	−0.20^*^	0.64^**^	-	
10	FFDI	−0.20^*^	−0.43^**^	−0.23^**^	−0.50^**^	−0.29^**^	−0.36^**^	−0.33^**^	0.65^**^	0.53^**^	-
11	PSI	−0.14	−0.38^**^	−0.23^*^	−0.47^**^	−0.27^**^	−0.24^**^	−0.35^**^	0.45^**^	0.55^**^	0.57^**^

* *p < 0.05*,

** *p < 0.01*.

*FFDI, Freedom from Distractibility; HI, Hyperactive-Impulsivity; IA, Inattention; POI, Perceptual Organization Index; Index; PSI, Processing Speed Index; VCI, Verbal Comprehension Index*.

Hierarchical regression analyses revealed significant contributions of age, gender, and ADHD diagnosis [Δ*R*^2^ = 0.082, Δ*F*_(3, 123)_ = 3.67, *p* = 0.01] to future peer problems at age 11 – 13. Not surprisingly, information about baseline peer problems added 18.5% to the explained variance [Δ*F*_(1, 122)_ = 30.83, *p* < 0.001]. Next, the IA and HI subscales added 4.5% to the explained variance [Δ*F*_(2, 120)_ = 4.17, *p* = 0.02], with the total model explaining 31.5% of the variance [*F*_(6, 120)_ = 9.19, *p* < 0.001].

To ascertain the relative contributions of IA and HI, we added each subscale in separate analyses (Pedhazur, 1982). When included in a third step beyond age, gender, ADHD status, and baseline peer problems, HI did not make a significant contribution [Δ*R*^2^ = 0.007, Δ*F*_(1, 121)_ = 1.21, *p* = 0.27], whereas IA did, increasing the model's explained variance by 4% [Δ*R*^2^ = 0.040, Δ*F*_(1, 120)_ = 7.07, *p* = 0.01]. Entering IA and HI scores rated by either parents or teachers separately did not alter the results. Inspection of the single predictors in the total model showed that only IA (*b* = 0.218, *SE* = 0.092, *p* = 0.017) and baseline peer problems (*b* = 0.405, *SE* = 0.124, *p* = 0.002) were significant in predicting future peer problems, when covarying the other variables.

Next, the contributions of age, gender, ADHD diagnosis, baseline peer problems, HI, IA, and the four WISC-III indices (VCI, POI, FFDI, and PSI) on future peer problems were analyzed using LARS, providing a reduced model including variables for the PROCESS analysis. LARS showed that the combination of baseline peer problems and IA in one step [Δ*R*^2^ = 0.308, Δ*F*_(2, 124)_ = 27.59, *p* < 0.001), and VCI and PSI in a later step [Δ*R*^2^ = 0.038, Δ*F*_(2, 122)_ = 3.54, *p* = 0.03] best explained variance in future peer problems, in total explaining 34.6% of variance [*F*_(4, 122)_ = 16.13, *p* < 0.001]. At the level of the individual variables, baseline peer problems, IA, and PSI (but not VCI) were significant predictors (Table 3).

**Table 3:** Baseline peer problems, inattention, and intellectual function as predictors of preadolescent peer problems.

	**B**	**SE**	***p***	**95% CI**
Baseline peer problems	0.412	0.120	0.001	0.180, 0.661
IA	0.132	0.058	0.024	0.022, 0.249
VCI	0.021	0.014	0.140	−0.006, 0.049
PSI	−0.027	0.011	0.018	−0.050, −0.003

*B, Beta Coefficient; CI, Confidence Interval; HI, Hyperactive-Impulsivity; IA, Inattention; PSI, Processing Speed Index; SE, Standard Error. VCI, Verbal Comprehension Index*.

**Figure 1:**
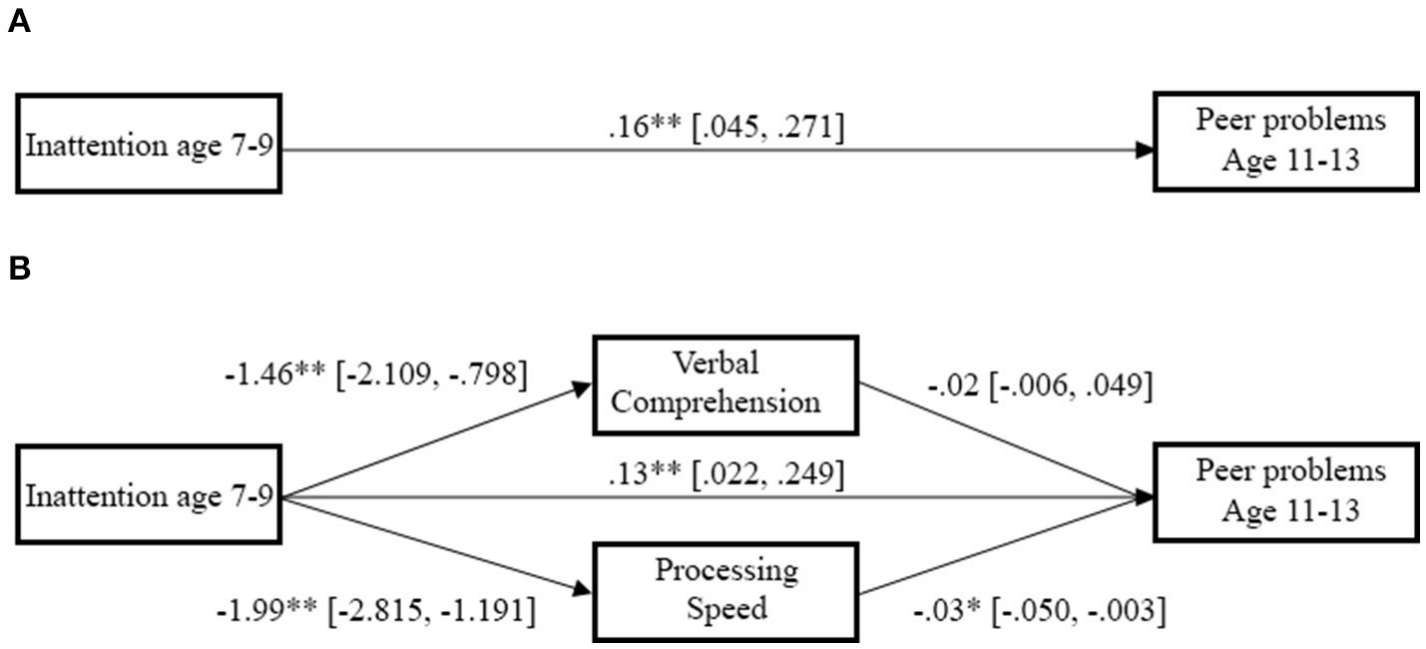
Direct and indirect relations between inattention, intellectual function and preadolescent peer problems. Lines represent the direct path between inattention and later peer problems, as well as the indirect path through Processing Speed and Verbal Comprehension, with numbers showing the bootstrapped point estimate with the 95% confidence interval in brackets. Inattentive symptoms were related both directly to later peer problems when controlling for PSI, VCI, and baseline peer problems, as well as by indirectly predicting reduced PSI performance and subsequent increased peer problems. ^*^p < 0.05, ^**^p < 0.01. **(A)** Direct path. **(B)** Mediated path.

The results from the LARS restricted the PROCESS analysis to include baseline peer problems as a covariate, IA as a direct predictor, and VCI and PSI as potential parallel mediators, with peer problems in preadolescence as the criterion variable. The PROCESS analysis showed a significant total effect for the baseline peer problems, IA, VCI, and PSI on peer problems in preadolescence [*b* = 0.155, *p* = 0.001, *95 CI* (0.065, 0.245)], with a significant direct effect of IA [*b* = 0.132, *p* = 0.009, 95 *CI* (0.033, 0.232)]. Mediation analyses showed that PSI was a significant mediator of the effect [*b* = 0.053, 95 *CI* (0.013, 0.110)], whereas VCI was non-significant [*b* = −0.031, 95 *CI* (−0.013, 0.075)] (see Figure 1). In short, the relation between childhood IA and future peer problems was partly explained through its impact on PSI, such that future peer problems were best explained by high IA scores in conjunction with low PSI performance. Importantly, these results remained after adjusting for the presence of an ADHD diagnosis.

## Discussion

Previous research has consistently linked ADHD to peer problems (Ros and Graziano, 2017), particularly in children with severe IA symptoms (Mikami et al., 2007). ADHD and IA have also been related to impaired intellectual function, with some studies emphasizing the importance of processing speed (Calhoun and Mayes, 2005; Thaler et al., 2013). The aim of the present longitudinal investigation was to examine associations between symptoms of IA and HI, different aspects of intellectual function, and future peer problems. The participants represent a clinically diverse sample derived from population sampling, and baseline peer problems and demographic variables were covaried. We found that peer problems in preadolescence were predicted by reports of ADHD related symptoms 3–4 years earlier. IA was found to be the strongest predictor, with PSI partially mediating its link to future peer problems. These findings remained significant even after including definite or probable ADHD diagnostic status as a covariate. These results confirm that IA and PSI in primary school children are linked to peer problems in adolescents, and support that their importance is not restricted to children with a specific diagnosis (Tseng et al., 2014).

Out of the four WISC-III indices, only PSI was identified as a significant predictor and mediator of later peer problems, despite significant correlations between IA and all WISC indices. Thus, our findings emphasize PSI's contribution to future peer problems, along with the value of considering specific intellectual indices in studies of functional outcomes (e.g., Keith and Witta, 1997). The importance of PSI may reflect that children with slow processing speed fail to adequately follow social interactions with their peers, potentially responding off-topic or socially disengaging if the social situations are perceived as an area of failure. For parallel findings, see Mikami et al. (2007), where, in a sample of children with ADHD-I, IA led to later peer difficulties because the inattentive child was perceived as a less entertaining playmate than other children. Some studies of children with coexisting conditions have also highlighted the importance of processing speed. For example, slower processing speed has previously been related to greater communication difficulties in individuals with high-functioning autism spectrum disorder (Oliveras-Rentas et al., 2012).

IA also predicted later peer problems when ADHD diagnosis was covaried, even though the IA scores were high in the ADHD group. This result suggests that IA may be important for both typically developing children and for those with ADHD, and fits previous research emphasizing the dimensional impact of ADHD symptoms (e.g., Kofler et al., 2011; Bunford et al., 2015). We therefore argue that ADHD symptoms below the diagnostic threshold should still be assessed and addressed in treatment, as they may both interfere with social development and cascade into larger problems over time (Tseng et al., 2014). Furthermore, the finding that HI was not significantly related to later peer problems corresponds to previous findings indicating that HI might be a less salient predictor for peer problems and intellectual function than IA (e.g., Chhabildas et al., 2001; Huang-Pollock et al., 2009; Mayes et al., 2009).

Processing or psychomotor speed has not been studied to the same extent as other aspects of intellectual function, but is nonetheless an important dimension in developmental neuropsychology (Kail and Hall, 1994; Waber et al., 2007). Furthermore, it has an important role in fluid intelligence and working memory (Fry and Hale, 2000), and low processing speed has been related to academic underachievement (Mayes and Calhoun, 2007). Our findings show that slow processing speed may be a risk factor for the future social functioning of a child, and thus constitutes a potential target for intervention. Although processing speed is reported to be unaffected by pharmacological treatment (Riordan et al., 1999; Biederman et al., 2008), a combination of medication and behavioral interventions can improve children's academic and homework efficiency (Jensen et al., 2007; Powers et al., 2008). Focused training directed toward organizational skills may also yield clinical benefit, potentially reducing the negative impact of low processing speed (Bikic et al., 2016).

## Limitations

Although the longitudinal design, inclusion of dimensional measures of ADHD symptom clusters, and assessment of intellectual functioning according to validated instruments comprise key main strengths of the present study, several limitations constrain the findings. First, we lacked sociometric measures for the assessment of children's real-life interactions with peers and peer reputations, which are considered the “gold standard” for appraisal of these variables. We also lacked complete data for teacher reports of peer problems to conduct the cross-informant analysis of peer problems strongly recommended by findings in previous studies (Renk and Phares, 2004). Second, additional measures of intellectual functioning could have provided even finer grained analyses. Third, repeated assessment of intellectual functioning was not included in the study design, and panel analysis including all variables at several time-points would have strengthened the inference of potential causal linkages.

## Conclusion

Findings from the present longitudinal study reveal that dimensions of IA, measured by teacher and parental reports, along with processing speed, are important factors in predicting peer problems in preadolescence, even when adjusting for prior peer problems. Mediation analyses confirmed the meaningful role of IA as well as its pathway through processing speed, linked to later peer problems 3–4 years later. The results remained significant after adjusting for ADHD status, showing that the results were not restricted to children with an ADHD diagnosis. A better understanding of the determinants of peer problems is needed to develop effective interventions procedures preventing development of social and other mental health problems later in life.

## Author contributions

AT was responsible for the data analysis, interpretation, drafting, and revising the manuscript. JM and SH contributed to drafting and revising the manuscript. AL was responsible for data collection in the Bergen Child Study, and contributed to the data analysis, interpretation, drafting, and revising the manuscript.

### Conflict of interest statement

The authors declare that the research was conducted in the absence of any commercial or financial relationships that could be construed as a potential conflict of interest.

## Supplementary material

Supplementary Figure 1 Distribution of mental disorders in the sample. Bars show the number of children meeting diagnostic criteria for mental disorders other than ADHD. The anxiety category includes specific phobia (eight with ADHD, 11 without ADHD), social phobia (one with ADHD, six without ADHD), generalized anxiety disorder (one with ADHD, one without ADHD), obsessive-compulsive disorder (one without ADHD), and panic disorder (one without ADHD). The mood category includes dysthymia (two without ADHD) and adjustment disorder (one without ADHD). Conduct disorders include oppositional defiant (seven with ADHD, four without ADHD) and conduct disorders (one with ADHD, one without ADHD). The TS/tic disorder category include Tourette's Syndrome (four with ADHD, two without ADHD) and tic disorders (four with ADHD, seven without ADHD), while the category for other disorders includes enuresis/encopresis (six with ADHD, two without ADHD), alcohol abuse (one without ADHD), and intellectual disability (one with ADHD, one without ADHD) and unspecified other disorder (four with ADHD).

Supplementary Table 1 Distribution of parental education.
