# Supplementary figures and images for: Processing Speed Mediates the Longitudinal Association between ADHD Symptoms and Preadolescent Peer Problems
Source: Front Psychol. 2018 Feb 13;8:2154. doi: 10.3389/fpsyg.2017.02154 (PMC5816923; doi:10.3389/fpsyg.2017.02154)

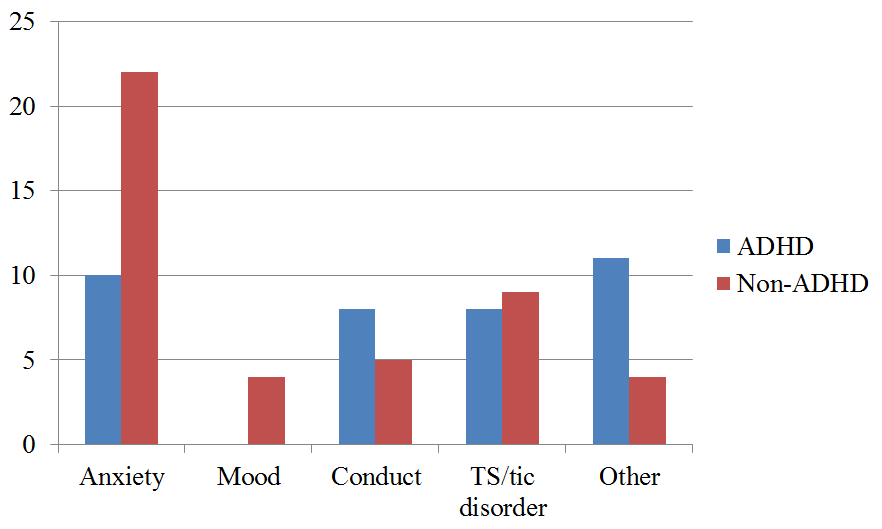

Supplement: Supplementary Figure 1 — Distribution of mental disorders in the sample. Bars show the number of children meeting diagnostic criteria for mental disorders other than ADHD. The anxiety category includes specific phobia (eight with ADHD, 11 without ADHD), social phobia (one with ADHD, six without ADHD), generalized anxiety disorder (one with ADHD, one without ADHD), obsessive-compulsive disorder (one without ADHD), and panic disorder (one without ADHD). The mood category includes dysthymia (two without ADHD) and adjustment disorder (one without ADHD). Conduct disorders include oppositional defiant (seven with ADHD, four without ADHD) and conduct disorders (one with ADHD, one without ADHD). The TS/tic disorder category include Tourette's Syndrome (four with ADHD, two without ADHD) and tic disorders (four with ADHD, seven without ADHD), while the category for other disorders includes enuresis/encopresis (six with ADHD, two without ADHD), alcohol abuse (one without ADHD), and intellectual disability (one with ADHD, one without ADHD) and unspecified other disorder (four with ADHD). [file Image1.TIF]
