# Supplementary material for: Processing Speed Mediates the Longitudinal Association between ADHD Symptoms and Preadolescent Peer Problems
Source: Front Psychol. 2018 Feb 13;8:2154. doi: 10.3389/fpsyg.2017.02154 (PMC5816923; doi:10.3389/fpsyg.2017.02154)
Supplement: Supplementary Table 1 — Distribution of parental education. [file Table1.docx]

Supplementary Table 1

*Distribution of parental education*

|  | n | %^b^ |
| --- | --- | --- |
| **Paternal education** |  |  |
| Lower secondary school | 13 | 10 |
| High school | 42 | 33 |
| College/university (up to four years) | 33 | 26 |
| College/university (more than four years) | 35 | 28 |
| **Maternal education** |  |  |
| Lower secondary school | 5 | 4 |
| High school | 44 | 35 |
| College/university (up to four years) | 40 | 31 |
| College/university (more than four years) | 38 | 30 |

**Note:** ^b^ Percentages for paternal education does not add up to 100 as four fathers did not report their education.
